# Enhancing intrauterine insemination success in advanced maternal age: Impact of consecutive ejaculate and optimised cycle parameters

**DOI:** 10.5935/1518-0557.20250195

**Published:** 2026

**Authors:** Gulam Bahadur, Roy Homburg, Ralf Henkel, Kanna Jayaprakasan, Santanu Acharya, Bryan J. Woodward, Asif Muneer, Judith A. F. Huirne, Abha Govind, Afeeza Illahibuccus, Ansam Al-Habib, Seang L.Tan, Eric Jauniaux

**Affiliations:** 1 Reproductive Medicine Unit, Royal Free Hospitals Trust, Sterling Way, London N18 1QX, UK; 2 Reproductive Medical Unit, University College London Hospital, London, NW1 2BU, UK; 3 LogixX Pharma Ltd., Theale, Berkshire, UK; 4 Department of Medical Bioscience, University of the Western Cape, Bellville, South Africa; 5 University Hospitals of Derby and Burton NHS Trust, Royal Derby Hospital, Derby, UK; 6 University Hospital Crosshouse-, Ayrshire Fertility Unit-, Kilmarnock- KA2 0BE- Scotland, UK; 7 X&Y Fertility, New Walk, Leicester, LE1 7JA, UK; 8 Department of Andrology and NIHR Biomedical Research Centre, University College London Hospital, London NW1 2PG, UK; 9 Amsterdam University Medical Centerslocation VUmc and AMC-, Amsterdam Research Institute Reproduction and Development-, De Boelelaan 1081-1081 HV Amsterdam, The Netherlands; 10 Department of ObGyn, McGill University, 845 Sherbrooke St W, Montreal, Quebec H3A 0G4 Canada; 11 EGA Institute for Women’s Health, Faculty of Population Health Science, University College London, London, WC1E 6HX, UK

**Keywords:** consecutive ejaculate, intrauterine insemination, IUI, male factor, women over 35 years

## Abstract

**Objective:**

This study evaluated whether consecutive ejaculate (CE) strategies improve intrauterine insemination (IUI) live birth rates (LBR) in women over 35 with unexplained or male-factor infertility. It also examined the influence of follicle number and sperm count thresholds on outcomes.

**Methods:**

In this retrospective cohort study (2010-2019), 596 IUI cycles were analysed in 263 nulliparous women-230 with CE and 366 with standard IUI. Among them, 98 patients underwent CE IUI and 165 received non-CE IUI. Patients with total motile sperm count (TMSC) <5×10^6^ were often fast-tracked to IVF, but CE was mostly attempted to boost sperm count beforehand. LBRs per cycle and per woman were compared between groups.

**Results:**

LBR per cycle was 11.3% (CE) vs. 13.1% (control) (*p*=0.52); per woman, 26.5% (CE) vs. 29.1% (control) (*p*=0.65). Mean ages were similar (37.7 vs. 38.0 years; *p*=0.34). Success improved with TMSC >10×10^6^: 65.4% (CE) and 87.5% (control). Over six cycles, LBR rose from 10.5% to 13.8% (CE) and 12.3% to 16.7% (control). Outcomes improved with two or three follicles, especially in women over 35.

**Conclusions:**

CE IUI yields LBRs comparable to standard IUI and may offer a cost-effective, less invasive alternative to IVF for male-factor infertility in women over 35. The LBRs per woman undergoing IUI were of a similar magnitude to those reported in IVF cycles. Optimising IUI LBR may involve increasing follicle numbers and using a higher TMSC threshold (>10×10^6^). CE IUI supports healthcare sustainability while expanding fertility treatment access.

## INTRODUCTION

Intrauterine insemination (IUI) is a minimally invasive and cost-effective first-line treatment for infertility, which affects approximately 1 in 6 couples worldwide (Cohlen *et al.*, 2018; Man *et al.*, 2023). IUI expands treatment options and enhances patient autonomy. Currently, fertility treatment primarily focuses on unexplained infertility, with positive pregnancy outcomes often linked to gonadotrophin stimulation and the presence of two follicles (Diamond *et al.*, 2015; Man *et al.*, 2023).

IUI procedures are typically guided by the arbitrary threshold of 5 million motile sperm per insemination, despite successful pregnancies reported with sperm counts below this figure (Merviel *et al.*, 2010; Man *et al.*, 2023). Meanwhile, male factor infertility has largely been overshadowed by the widespread use of the more expensive IVF (IVF-ICSI) procedures. The adoption of IUI is hindered by commercial and economic incentives that prioritise costly ICSI procedures, despite ICSI offering no additional benefit in IVF cases with normozoospermia, while being potentially associated with increased foetal risks (Hansen *et al.*, 2002; Dang *et al.*, 2021). The rapid expansion of IVF has contributed to stagnation in male fertility management, with limited efforts to optimise IUI. Although substantial evidence supports IUI as an effective first-line treatment before IVF, arguments favouring IVF over IUI persist (Guideline Group on Unexplained Infertility, 2023; Lai *et al.*, 2024). Male factor infertility guidelines generally advocate for less invasive and more accessible treatments, such as IUI over IVF, but fail to provide clear guidance on sperm quality criteria for selecting an appropriate management approach (Brannigan *et al.*, 2024).

IVF is often viewed as a quick fix for subfertility, but this perception is misleading, as around 70% of women remain barren after IVF treatment (HFEA, 2022). Success rates for IVF seem to be driven by the treatment of patients who could have potentially conceived through IUI. If IUI were utilized more widely, it could free up IVF resources for those who genuinely need it, while addressing the economic challenges associated with the high neonatal care costs and increased risk of multiple pregnancies and fetoplacental abnormalities in IVF conceptions (Hansen *et al.*, 2002; Wen *et al.*, 2012; Bahadur *et al.*, 2020). IUI also serves as a useful tool for IVF practitioners to preserve success rates, particularly when dealing with difficult cases (Quinquin *et al.*, 2014; Delbos *et al.*, 2018). There is significant global potential for IUI to improve patient access to fertility treatments, particularly in resource-limited settings, making it a cost-effective strategy for governments and healthcare agencies (Bahadur *et al.*, 2020).

In a previous pilot study, we found that CE (Male factor Cohort) strategy in couples where the male partner does not meet the 5 million motile sperm thresholds for insemination reduces the need IVF referrals (Bahadur *et al.*, 2016). The aim of the present study is to evaluate whether CE strategy is a viable option with IUI in women over 35 years in cases that would otherwise be fast-tracked to IVF.

## MATERIALS AND METHODS

### Study Population

This is a single-centre retrospective observational study at a UK Hospital Trust (Royal Free Hospital NHS Foundation Trust, formerly North Middlesex University Hospital NHS Foundation Trust) specialising in IUI procedures, with referrals to NHS IVF clinics where required. Patients are offered up to six IUI cycles.

We have analysed IUI cycles performed between 2010 and 2019 in couples where the female partner was over 35 years of age. A total of 596 IUI cycles were reviewed in 263 patients, including 230 cycles undergoing IUI with CE treatment and 366 standard (non-CE) control (unexplained infertility) treatments, performed in 98 and 165 patients, respectively. The women’s age refers to the age at the time of IUI and the associated outcomes.

### Inclusion criteria

Nulliparous women in heterosexual relationships with over one year of subfertility; women with previous failed IVF cycles.

### Exclusion criteria

History of cancer; tubal disease or occlusion identified via hysterosalpingogram; reversal of sterilization; multiparous women, use of donor semen.

### Scheduling and Operator Consistency

Due to resource constraints, inseminations were limited to a three-day alternate weekday schedule. However, consistency in operators ensured uniformity. Scheduling limitations were acknowledged as a potential confounding factor and analysed accordingly in this study.

### Management protocol

All patients underwent a standard protocol using gonadotropin stimulation with 150 IU of Menopur (Ferring, Kiel, Germany) administered on alternate days, with dose adjustments based on follicular response. Ovulation was triggered ideally 29 hours before IUI using human chorionic gonadotropin (hCG) (Pregnyl, Gonasi or Ovitrelle). Luteal support was provided with Cyclogest (400 mg vaginal pessaries) administered twice daily for 14 days post-IUI.

Ovarian hyperstimulation syndrome (OHSS) risk minimization and multiple birth assessments were carefully managed. Cycles were abandoned in cases of: OHSS development; presence of four or more leading follicles; lack of response despite gonadotropin dose adjustments.

Women with two to three follicles were considered on a case-by-case basis for the risk of multiple gestation pregnancy (MGP). Follicles up to 18-19 mm were considered in the MGP risk assessment for cycle cancellation, while smaller follicles of up to 14 mm were factored into risk assessments for IUI MGP risks.

When the leading follicle(s) reached 17-18 mm, ovulation was triggered using either Pregnyl/Gonasi (5000 IU) or Ovitrelle (choriogonadotropin alfa, 250 µg, Merck Sharp & Dohme Ltd). IUI was scheduled ideally 29 hours post-trigger, but within a range of 24-40 hours. If ovulation was confirmed (presence of corpus luteum and free fluid in the pelvis), insemination occurred on the same day. Sexual intercourse post-IUI was supported if no added risk factors for MGP were identified.

Semen samples were processed and prepared immediately before insemination. The Rocket EDL IUI Catheter R57622 was used for insemination, followed by 15 minutes of bed rest.

### Follow-Up and Outcome Measures

Post-insemination, patients continued progesterone supplementation and were advised to take a pregnancy test after 14 days. Pregnancy was confirmed by detecting a foetal heartbeat, and any adverse outcomes were monitored through to live birth (LB).

The Human Fertilisation and Embryology Authority (HFEA) requires the submission of pregnancy rates per cycle by the end of February for the previous calendar year, with miscarriages excluded from these calculations. Therefore, HFEA data published on the website should be interpreted as reflecting only live birth rate (LBR), as provisions are made to report late-stage miscarriages from pregnancies that extend to the end of the calendar year. Our data reflects only the LBR.

### Sperm characteristics

In male patients with oligozoospermia, where the initial total motile sperm count (TMSC) was <10×10^6^ sperm before processing, a CE was requested within 30 minutes after the first ejaculate to supplement the initial sample. Patients with a TMSC below 5×10^6^ sperm at the time of consultation and evaluation were typically referred to IVF clinics.

To optimise IUI success, patients with a potential motile sperm deficit (<5×10^6^ sperm) were encouraged to provide a CE to assess their ability to produce a second ejaculate for sperm-washing procedures. Sperm preparation for CE focused on maximising motile sperm recovery, often retrieving motile sperm from both washings. This process could extend sperm processing time.

There was a recognised risk of not obtaining the required TMSC on the day of the IUI. However, the procedure was not cancelled solely due to a low TMSC. For successful CE production, male patients were provided with counselling and support to build confidence. To alleviate performance anxiety, some flexibility was offered, allowing the CE to be produced within a 30-minute window after the first ejaculate. At this stage, all samples were accepted, and even low sperm numbers in the CE were considered beneficial for insemination. For IUI treatment purposes, only the final TMSC per insemination was recorded. Most men had no difficulty producing a CE.

Sperm preparation was performed using density gradient centrifugation with PureCeption (Sage, Trumbull, CT, USA, and Origio Ltd, UK) and Quinn’s Sperm Washing Medium (Sage, Cooper Surgical Ltd).

### Statistical analysis

The primary outcome was a comparison of LBR in women who were inseminated with CE versus the standard single ejaculate insemination method for unexplained infertility (control). The secondary outcome was to investigate and describe any factors that were associated with a positive LB. A cancelled cycle following ovarian stimulation is regarded as a cycle started and is included in the overall calculations.

The analysis of the data was carried out using MedCalc^®^ Statistical Software version 23.1.7 (MedCalc Software Ltd, Ostend, Belgium). Data was tested for normal distribution using the Shapiro-Wilk test. Depending on the distribution, further analyses were done using the Chi-squared test, t-test, Mann-Whitney test, and Kruskal-Wallis test with the Jonckheere-Terpstra test for trend analysis. In addition, odds ratios were calculated. The data are presented in means and standard deviation, or percentages. A *p*-value of *p*<0.05 was considered significant.

## RESULTS

Out of a total of 596 couples participating in the study, 230 were assigned to the CE (Male factor Cohort) and 366 to the control group (Unexplained infertility cohort). No differences between the CE and control groups were found for the female age at the time of the IUI (Table 1).

**Table 1:** Comparison of female age at IUI and at pregnancy, and the total motile sperm count at IUI. There was no difference between the study group CE (Male factor group) and the Control (Unexplained Group).

Variable	Consecutive Ejaculate Group	Control	p-value
Age at IUI (y)	37.7±1.6 (n=230 cycles)	38.8±2.0 (n=366 cycles)	0.3433 (Mann-Whitney test)
Age at pregnancy (y)	37.2±1.7 (n=26 patients)	37.7±1.7 (n=48 patients)	0.2672 (independent t-test)
Total motile sperm count (10^6^) at IUI	14.9±7.6 (n=26 patients)	16.4±7.2 (n=48 patients)	0.4412 (Mann-Whitney test)

While 26 LB in 98 women (26.5%) were recorded in the CE group, 48 women out of 165 (29.1%) in the control group conceived (*p*=0.65). The LBR/cycle were 11.3% (CE group) and 13.1% (control group), respectively, and did not differ (*p*=0.67). The highest LBR occurred when the TMSC exceeded 10×10^6^ sperm, with overall success rates of 65.4% (17 out of 26) in the CE and 87.5% (42 out of 48) in the control group. The age distribution with the number of LB achieved in both study groups is depicted in Table 2. When we compared the number of LB over 6 cycles (Table 3), no statistically significant difference between the first 3 cycles and the remaining 3 cycles was observed for the CE group (*p*=0.49) with an odds ratio of 0.73 (*p*=0.49) and in the control group (*p*=0.34) with an odds ratio of 0.70 (*p*=0.35). When comparing the LBR/cycle in the first 3 cycles in the CE group with that of the first 3 cycles in the control group, there was also no difference (*p*=0.56). A similar result was observed for the second 3 cycles (cycle number 4 to 6) (*p*=0.66). The odds ratios are shown in Table 3.

**Table 2:** Distribution of patient ages and numbers of live births achieved in the consecutive ejaculate and control groups.

Age at IUI (y)	Consecutive Ejaculate Group (n=98 patients)		Control (n=165 patients)	
	Number of cycles	Live births	Number of cycles	Live births
35	41	8	51	9
36	32	6	85	11
37	52	4	70	10
38	40	6	66	7
39	42	3	36	5
40	8	0	27	5
41-43.6	7	1	31	1
Total	230	26	366	48

**Table 3:** Comparison of the Number of Live Births over 6 Cycles.

Consecutive Ejaculate Group (Male factor)							Control Group (Unexplained factor)							Comparison of Live Birth between first 3 cycles and next 3 cycles
**Cycle**	**Total number of cycles**	**Number of live births**	**Combined number of Cycles**	**Live births in cycles combined**	**Live births/** **cycle**	**Odds** **ratio** **(p)**	**Cycle**	**Total number of cycles**	**Number of live births**	**Combined number of Cycles**	**Live births in cycles combined**	**Live births/cycle**	**Odds** **ratio** **(p)**	**Consecutive Ejaculate** **vs** **Control** **Odds ratio (p)**
1	67	8	172	18	10.5%	0.7305 (0.4902)	1	146	15	300	37	12.3%	0.7034 (0.3470)	0.8308 (0.5431)
2	62	8					2	97	14					
3	43	2					3	57	8					
4	27	1	58	8	13.8		4	38	5	66	11	16.7%		0.8000 (0.6580)
5	22	5					5	16	4					
6	9	2					6	12	2					

**Figure 1:**
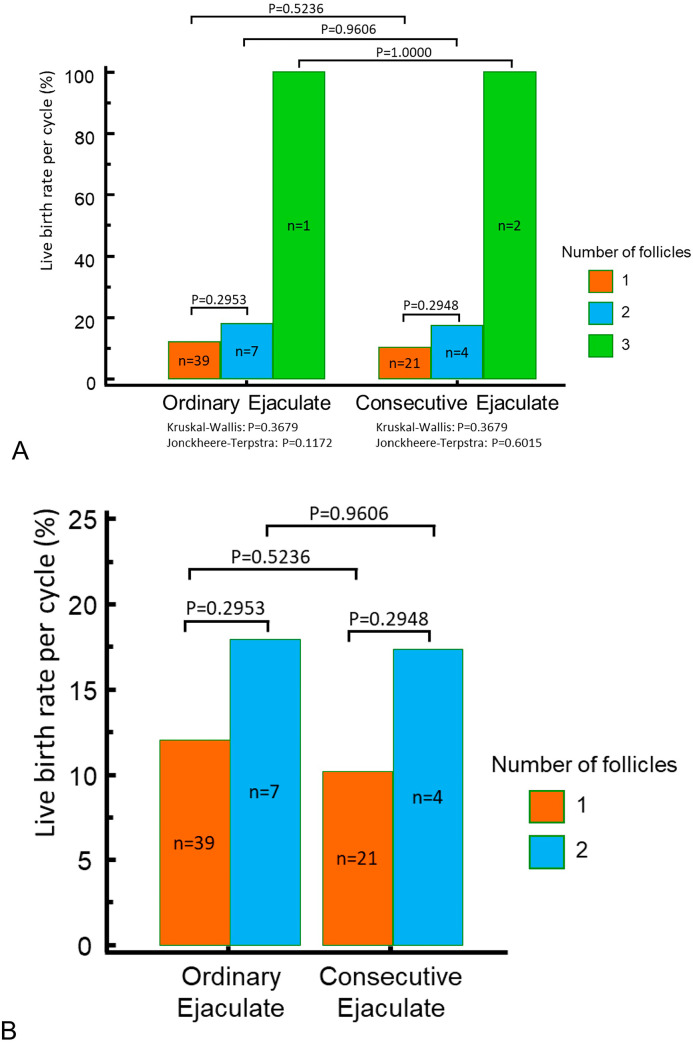
Comparison of live birth rates per cycle according to the number of follicles observed. Distribution of live birth rates among 3 (A) and 2 (B) follicles. No difference between the groups can be seen.

Although the LBR/cycle with 2 follicles were higher than when there only one follicle (Figure 1, Table 4), no difference between 1 and 2 follicles was found in the CE group (*p*=0.29) and the control group (*p*=0.30). When both study groups were compared, there were also no differences for patients with 1 follicle (*p*=0.5236) and those with 2 follicles (*p*=0.96), respectively. Considering that only 1 and 2 LB in the CE and control group, respectively, were achieved, resulting in LBR/cycle of 100% in each group, 3 follicles were not considered for further calculations (Figure 1). These results are confirmed in the Kruskal-Wallis and Jonckheere-Terpstra tests indicating no trend toward higher LBR if more follicles are present. In a total of 3 women, 3 follicles developed (CE group: 1 woman; control group: 2 women). Therefore, the inclusion of these few cases into the calculation is rather distorting the results should be treated with caution (Figure 1). For the odds ratios, similar results were obtained (Table 4).

**Table 4:** Comparison of live birth rate per cycle between the CE and control group for the number of follicles identified in the ultrasound. No differences between different comparison groups were found for the odds ratios.

Consecutive Ejaculate Group					Control					Comparison
**Follicles**	**Cycles**	**Live births**	**Live births/cycle**	**Odds ratio** **(*p*)**	**Follicles**	**Cyclse**	**Live births**	**Live births/cycle**	**Odds ratio** **(*p*)**	**Consecutive Ejaculate** ***vs*.** **Control** **Odds ratio** **(*p*)**
1	206	21	10.2%	0.5392 (0.3003)	1	235	39	12.0%	0.6234 (0.2945)	0.8324 (0.5223)
2	23	4	17.4%		2	39	7	17.9%		0.9624 (0.9557)
3	1	1	100%		3	2	2	100%		

**Figure 2:**
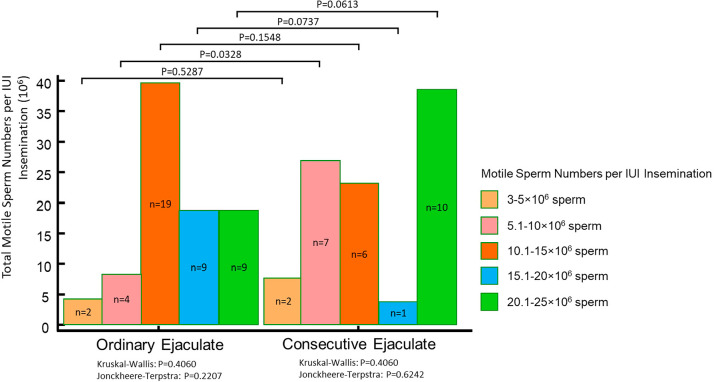
Relationship between total motile sperm counts and live births after IUI in consecutive ejaculate and control cohorts. No differences between the different groups can be seen.

Similarly, Kruskal-Wallis and Jonckheere-Terpstra tests did not reveal any trend toward higher LBR if higher total motile sperm counts were used in the CE group (*p*=0.41 and *p*=0.62, respectively) and the control group (*p*=0.41 and *p*=0.22, respectively). The marginal and borderline significant differences in the TMSC used for insemination between the CE and control groups appear to be due to the small sample sizes (Table 5, Figure 2).

**Table 5:** Comparison of the live birth rates for different categories of the total motile sperm counts used for the IUI.

Consecutive Ejaculate Group			Control			Comparison	
**Total Motile sperm count (10^6^)**	**Live births**	**Live births / cycle**	**Total Motile sperm count (10^6^)**	**Live births**	**Live births / cycle**	**Odds ratio**	**p-value**
3-4.5	2	7.7%	3-4.8	2	4.2%	1.8462	0.5514
5-9.9	7	26.9%	6.4-8.1	4	8.3%	3.2308	0.0812
10-14.9	6	23.1%	10.4-14.8	19	39.6%	0.5830	0.3067
15-19.9	1	3.8%	15.3-19.6	9	18.8%	0.2051	0.1431
20-25.6	10	38.5%	20-24	9	18.8%	2.0513	0.1671
			24-28.8	3	6.3%		
			30.2-41.6	2	4.2%		
Number of live births	26			48			

Another confounding factor that could possibly affect the onset of LB after IUI is the weekday of insemination as the insemination procedure at our hospital was restricted to a 3-day alternate weekday due to resource constraints. Results, however, show no difference between the two study groups for Monday, Wednesday and Friday as days of insemination. Within each study group, no difference in the LBR could be seen between CE group (Mon-Wed: *p*=0.54; Wed-Fri: *p*=0.26; Mon-Fri: *p*=0.08). In the control group, except for Mon-Wed (*p*=0.02), no differences were found for the other weekdays (Wed-Fri: *p*=0.41; Mon-Fri: *p*=0.11) (Table 6). Hence, results reflect the dedicated efforts of clinical, nursing, and scientific staff, though operational constraints must be considered in broader applications. In addition, except for 1 MGP with a monochorionic diamniotic twin in the CE cohort, we did not observe any other adverse circumstances in this study to warrant cancellation of a cycle.

**Table 6:** Effect of weekend management on live birth rates after IUI. No negative influence of the 3-day alternate schedule on the live birth rates can be seen.

Consecutive Ejaculate Group		Control		Odds ratio (*p*)
Day	Live births	Day	Live births	
Monday	6	Monday	10	1.1077 (0.8578)
Wednesday	8	Wednesday	21	0.7033 (0.4648)
Friday	12	Friday	17	1.3032 (0.5552)
Total number of live births	26	Total number of live births	48	

## DISCUSSION

The results of the present study indicate that a similar LBR can be achieved with IUI for male factor infertility when combined with CE samples compared with single ejaculate samples in the unexplained infertility group (Control cohort), thereby obviating referral to IVF procedures due to low sperm counts. Furthermore, the overall clinical management and scientific approach to the control and CE groups are similar in terms of IUI LBRs with those reported for IVF procedures (HFEA, 2022). Investigating a subgroup of couples where the female partner’s age was over 35 allowed us to minimise variables associated with younger age groups and concentrate on key factors that may contribute to improved success rates. These include the use of CE in cases where otherwise male factor infertility would typically directly lead to IVF, the number of treatment cycles, follicle count, total motile sperm count (TMSC), and whether limiting IUIs to alternate weekdays may compromise patient outcomes.

Since the publication of a pilot study on the use of CE in IUI cycles (Bahadur *et al.*, 2016), there has been growing interest in CE sperm kinematics (Alipour *et al.*, 2017) and the associated reduction in sperm DNA fragmentation in the sperm available (Karavani *et al.*, 2023). Both parameters are correlated with improved pregnancy rates and reduced miscarriage rates, without additional treatment interventions (Barbagallo *et al.*, 2022; Repalle *et al.*, 2022). The present findings indicate a LBR per cycle of 11.3% in the CE group and 13.1% in the control group (*p*=0.52; Table 1). The LBR per woman was 26.5% in the CE group and 29.1% in the control group (*p*=0.65), with both groups achieving LBRs comparable to those seen with IVF (Bahadur *et al.*, 2020; HFEA, 2022). The justification for six IUI cycles in the present study is supported by the observation that all six cycles were beneficial. The recommended number of IUI cycles is generally set at six (Tjon-Kon-Fat *et al.*, 2015). In our cohort, there were no significant differences in success rates between the first three IUI cycles and the second three IUI cycles (Table 2). The LBR for cycles 1-3 *versus* 4-6 was 10.5% *vs*. 13.8% for the CE group (OR: 0.73; *p*=0.49) and 12.3% *vs*. 16.7% for the control group (OR: 0.70; *p*=0.35). However, identifying women least likely to conceive after six cycles could help avoid negatively impacting overall success rates.

The number of follicles is crucial in determining LBR success but must be balanced against the risk of MGP. Comparing success rates between cycles with one *versus* two follicles, the LBR per cycle increased from 10.2% to 17.4% (*p*=0.29) in the CE group and from 12.0% to 17.9% (*p*=0.30) in the control group (Table 4, Figure 1). Although the increase was not statistically significant, the data suggest that having two follicles-and possibly three-may improve LB outcomes, particularly for women over 35, who had individualised MGP risk assessments. However, the small sample size for cycles with two or three follicles limits definitive conclusions, underscoring the need for larger studies. Notably, one monochorionic diamniotic twin LB occurred in a 35.6-year-old patient in the unifollicular CE cohort undergoing her first IUI cycle, with a total motile sperm count (TMSC) of 12.6×10^6^. Increasing IUI follicles from one to two may triple pregnancy chances (Tomlinson *et al.*, 1996); while elevated oestradiol levels (>1000 pg/mL) during induction may give a clue to avoiding MGP risks, as this level is associated with fivefold increased risk of triplets (Dickey *et al.*, 2001). Our data indicated that in women over 35 years old that having two or three follicles in IUI is associated with a low risk of MGP. Three follicles may offer improved success rates without a significant increase in MGP risk, especially compared to transferring three embryos in IVF, where foetal reduction procedures are more common (Bahadur *et al.*, 2020). However, for women under 35, where MGP risk is higher, stricter cancellation policies may be more appropriate. Importantly, women over 40 may benefit from having up to three follicles, as this provides a realistic chance of LB success without excessive risk (Table 2 and 4).

The commonly cited threshold for motile sperm counts in successful IUI-5×10^6^ sperm-may require reassessment as it may not be optimal (Khalil *et al.*, 2001). In our cohort, the highest LBR was observed when motile sperm counts exceeded 10×10^6^ sperm, with success rates of 65.4% in the CE group and 87.5% in the control group (Table 5, Figure 2). These findings suggest that IUI policies should be reconsidered, as higher sperm concentrations may significantly improve LB outcomes. This aligns with emerging evidence indicating that a threshold of >10×10^6^motile sperm per IUI may yield superior results (Van Voorhis *et al.*, 2001). Although pregnancies are possible with sperm counts below 5×10^6^ sperm, increasing the number of follicles may help overcome this limitation and enhance success rates.

Another factor that may have influenced success rates in our cohort was the alternate weekday scheduling of IUI procedures. While there was no indication that IUIs performed on Mondays negatively impacted outcomes, those conducted on Fridays showed slightly better-though not statistically significant-results (Table 6). In contrast, the control group exhibited significantly higher success rates for IUIs performed on Wednesdays, a pattern not observed in the CE group. Overall, there was no evidence that the specific day of IUI influenced outcomes within the CE group. However, due to the limited sample sizes, comparisons across subgroups should be interpreted with caution.

The strengths of this study included that it was a single-centre investigation dedicated to the IUI procedure, with a reasonably large patient sample. Notably, there were no cancellations due to the failure to produce a CE, which is a positive aspect of the study design. It also represents the first indication of expanding the scope of IUI to address potential male factor infertility. In many cases, women with male factor infertility may have a higher chance of conception if the man is properly diagnosed and sufficient sperm are available at the right time. Furthermore, this approach allowed for the potential to increase follicle numbers, compensating for male factor issues and the impact of female age on fertility.

On the other hand, the retrospective design of our study is associated with inherent limitations. The IUI protocol was limited to a three-day timeframe and sperm parameters were only assessed based on total progressive sperm count due to constraints in the ability to collect initial parameter data as one would in a randomized controlled trial. Additionally, profiling patients for their ability to produce CE within half an hour for sperm preparation purpose, prior to IUI may not always be reproducible on the day of the procedure and could therefore affect consistency. Moreover, while the sample size in this study was reasonable, it was not large enough to definitively generalise the findings. In addition, ovarian reserve data for women over 35 was not available in our current database and therefore limited the ability to draw conclusions in this age group. Associated DNA fragmentation correlations between CE and control arms would be of scientific interest as the clinical benefits of shorter abstinence are being widely recognised for favourable lowered DNA fragmentation levels (Barbagallo *et al.*, 2022; Repalle *et al.*, 2022; Karavani *et al.*, 2023).

The male factor was based on the ability to provide 5×10^6^ progressively motile sperm for IUI insemination at the point of clinical consultation. Prior to this innovation, the policy was to fast-track patients to IVF treatment. In this study, patients scheduled for IUI treatment were not cancelled if this sperm threshold was not met on the day of the scheduled IUI. The success rates, as measured per cycle and per woman, suggest that the outcomes could mirror the levels of success achieved in IVF procedures. Our data remains unique in the IUI field.

IUI is recognised as one of the top three global research priorities in assisted reproduction (Duffy *et al.*, 2021). However, its development is often constrained by the IVF sector’s focus on more complex procedures (Duffy *et al.*, 2021). Male factor infertility, commonly overlooked due to the routine use of ICSI (Dang *et al.*, 2021), continues to receive less research funding and public health focus than female reproductive health (Brannigan *et al.*, 2024; De Jonge *et al.*, 2024). Although strong evidence supports IUI as the first-line treatment for unexplained infertility, male factor cases are frequently fast-tracked to ICSI without comprehensive evaluation (Cohlen *et al.*, 2018; Guideline Group on Unexplained Infertility, 2023; Man *et al.*, 2023; Lai *et al.*, 2024). Given IUI’s advantages-patient acceptability, simplicity, and cost-effectiveness (Bahadur *et al.*, 2020) -our efforts have centred on optimising outcomes by identifying key contributing factors (Merviel *et al.*, 2010) and applying targeted strategies to enhance success rates. The use of CE has proven effective in increasing motile sperm counts among men with poor semen quality-individuals who might otherwise be prematurely referred to IVF without further assessment.

IUI offers substantial cost savings, with each live birth costing £42,558 less than IVF-and up to £76,257 with modest improvements in success rates (Bahadur *et al.*, 2020). IVF would require a 2.7% success rate increase for every 1% gain in IUI to match its cost-effectiveness. These figures exclude additional neonatal care and higher abnormality risks associated with IVF (Hansen *et al.*, 2002; Wen *et al.*, 2012; Bahadur *et al.*, 2020). Given these findings, there is a strong case for governments and policymakers to reinvest in IUI programmes and optimise their success rates. Expanding access to IUI would not only offer patients more treatment options but also significantly reduce costs for both individuals and healthcare systems. Our study highlights the potential of IUI in cases involving male factor infertility, further broadening its applicability and benefits. By prioritising IUI where appropriate, funding could be more effectively allocated-ensuring IVF is reserved for cases that genuinely require it and supporting better diagnosis of male infertility. This approach enhances patient outcomes, improves cost-efficiency, and reduces unnecessary medical risks associated with IVF. A re-evaluation of fertility policies is therefore urgently needed to ensure recommendations remain evidence-based, patient-centred, and economically sound. A key barrier to achieving this is the lack of accurate, balanced information. Due to the financial incentives surrounding IVF, IUI is frequently overlooked by practitioners despite its competitive success-particularly when comparing six IUI cycles to three IVF cycles. Many patients are unaware of this lower-cost option, limiting informed decision-making. To address this, we present success rates both per cycle and per woman, allowing for clearer, fairer comparisons between fertility treatments.

Few studies directly compare IUI and IVF outcomes (Bahadur *et al.*, 2020; Man *et al.*, 2023; Lai *et al.*, 2024), yet IVF is frequently favoured in policy without strong justification. IVF success is often reported using cumulative or survival analyses (Daya, 2005; Wang, 2006), which can mislead patients by presenting overly optimistic outcomes. The 2010 UK NICE guidelines, though not evidence-based, advised bypassing IUI, prompting a shift in practice and withdrawal of IUI funding. Interestingly, live birth rates per frozen embryo transfer rose from 12% in 2010 to 28% by 2020, despite no major IVF advancements in that time (HFEA, 2022) -suggesting many IVF cases may have been suitable for IUI.

Concerns around IUI-related MGP are often rooted in outdated data. Dickey *et al.* (2005) reported high-risk IUI cycles with up to 9 follicles and little oversight. In contrast, Evans *et al.* (2020) analysed 50,473 cycles under safer, modern protocols (1-5 follicles), yet policies continue to cite outdated risks, undermining IUI’s viability. The idea that fast-tracking patients to IVF improves outcomes lacks evidence (Guideline Group on Unexplained Infertility, 2023; Man *et al.*, 2023; Lai *et al.*, 2024). IVF marketing often pressures patients into three-cycle packages, citing inflated cumulative success rates. While some studies suggest over 70% success after three IVF cycles (Daya, 2005; Wang, 2006), real-world outcomes show only 30% of patients take home a baby (HFEA, 2022). Nearly 70% remain childless-similar to IUI outcomes-highlighting the cost-effectiveness of IUI (Bahadur *et al.*, 2020). Yet this cumulative failure rate is rarely disclosed, limiting patients’ ability to consider IUI as a viable, informed alternative.

In summary, optimised IUI practices, including the groundbreaking use of CE in male factor infertility, achieves LBR comparable to those in unexplained infertility and national IVF per embryo transfer data. Effective cycle management-targeting optimal follicle numbers and total motile sperm count (TMSC)-is central to success in gonadotrophin-stimulated IUI. This cost-effective, less invasive approach offers a viable first-line alternative to IVF/ICSI, particularly in resource-limited settings. It also addresses concerns related to multiple gestation pregnancy (MGP) and maternal-neonatal risks. Accurate patient counselling and prioritisation of IUI optimisation can expand access, reduce unnecessary IVF use, and align with evidence-based, patient-centred fertility care.

## CONCLUSION

This observational, non-randomised study uniquely demonstrates that patients typically fast-tracked to IVF due to low sperm counts can achieve comparable success rates with IUI when using CEs. For women over 35, routinely increasing follicle numbers to two or three should be considered, provided thorough risk assessments are conducted. We also recommend raising the total motile sperm count (TMSC) threshold from 5×10^6^ to 10×10^6^, though lower TMSC levels may still yield acceptable outcomes. Offering up to six IUI cycles as standard practice is justified, supported by consistent success rates across early and later cycles. With limited data further evaluation of weekend IUI management is warranted.

When optimised, IUI with CE offers a cost-effective, accessible alternative to IVF, with comparable per-woman success rates. A well-structured IUI program supports healthcare sustainability and empowers patients with greater autonomy and informed evidence-based choices. Presenting success rates per cycle and per woman enables clearer, more personalised decisions.

Most importantly, while IUI is typically used for unexplained infertility, inclusion of CE expands its scope-making it a viable option for some male factor infertility, offering a less invasive alternative to IVF, representing a meaningful shift in first-line fertility care.
